# Multi-omics analysis of fecal samples in colorectal cancer Egyptians patients: a pilot study

**DOI:** 10.1186/s12866-023-02991-x

**Published:** 2023-08-29

**Authors:** Randa H. Khattab, Rana H. Abo-Hammam, Mohammed Salah, Amro M. Hanora, Sarah Shabayek, Samira Zakeer

**Affiliations:** 1 Department of Microbiology and Immunology, Al-Salam University, Tanta, Egypt; 2 Forensic toxicologist and narcotics expert, Ministry of Justice, Tanta, Egypt; 3 Department of Microbiology and Immunology, Faculty of pharmacy, Port-Said University, Port-Said, Egypt; 4 Department of Microbiology and Immunology, Faculty of pharmacy, Suez Canal University, Ismailia, Egypt

**Keywords:** CRC, Gut microbiota, Metagenomic, Metaproteomic, Metabolomic, Tumor marker, Diagnostic biomarker.

## Abstract

**Background:**

Colorectal cancer (CRC) is a public health concern and the second most common disease worldwide. This is due to genetic coding and is influenced by environmental aspects, in which the gut microbiota plays a significant role. The purpose of this study was to compare the microbiota makeup of CRC patients with that of healthy control and to identify upregulated and downregulated proteins and metabolites in CRC patients. Using a next-generation sequencing approach, fecal samples of five females (4 CRC patients and one healthy control) were analyzed by BGI DNBSEQ-T7, Hong Kong, China. Furthermore, proteomics and metabolomics analysis were performed using LC-MS/MS technique.

**Results:**

Dysbiosis of gut microbiota has been observed in patients with CRC, with an increase in microbiota diversity at all taxonomic levels relative to healthy control. Where, at the functional level the bacterial species participate in many different pathways among them de novo nucleotide synthesis and amino acids pathways were aberrantly upregulated in CRC patients. Proteomics and metabolomics profiles of CRC patients showed different proteins and metabolites, a total of 360 and 158 proteins and metabolites, respectively were highly expressed compared to healthy control with fold change ≥ 1.2. Among the highly expressed proteins were transketolase, sushi domain-containing protein, sulfide quinone oxidoreductase protein, AAA family ATPase protein, carbonic anhydrase, IgG Fc-binding protein, nucleoside diphosphate kinase protein, arylsulfatase, alkaline phosphatase protein, phosphoglycerate kinase, protein kinase domain-containing protein, non-specific serine/threonine protein kinase, Acyl-CoA synthetase and EF-hand domain-containing protein. Some of the differential metabolites, Taurine, Taurocholic acid, 7-ketodeoxycholic acid, Glycochenodeoxycholic acid, Glycocholic acid, and Taurochenodeoxycholic acid that belong to bile acids metabolites.

**Conclusions:**

Some bacterial species, proteins, and metabolites could be used as diagnostic biomarkers for CRC. Our study paves an insight into using multi-omics technology to address the relationship between gut microbiota and CRC.

**Supplementary Information:**

The online version contains supplementary material available at 10.1186/s12866-023-02991-x.

## Background

Colorectal cancer (CRC) is one of the healthcare challenges and the second leading cause of cancer-related death worldwide [1]. It is related to genetic encoding [2] and more significantly, influenced by environmental aspects through a couple of years, the intestinal microbiota is an important contributor in addition to diets, lifestyle, and metabolic syndrome [3].

The Microbiome of the human gut is composed mainly of bacteria, viruses, and fungal organisms; in healthy conditions intestinal microbiota plays an essential role in human harvesting energy, intestinal epithelium determinative, pathogens protection, and immunity maintenance [4–8]. The interaction of the intestinal microbiota with the individual immune system is closely related, this can lead to continuous low-grade inflammation resulting in carcinogenesis. Contrariwise, inflammation only cannot induce CRC without the microbiota or compounds derived from bacteria [9].

CRC is treated in accordance with the stage of the cancer. The first line of treatment for early-stage CRC patients with localized disease is surgical resection. However, the majority of patients develop metastasis; A very common approach treatment of metastatic CRC includes a combination of chemotherapy agents, FOLFOX (5-FU, leucovorin, and oxaliplatin) or FOLFIRI (5-FU, leucovorin, and irinotecan), with biologic agents, targeted monoclonal antibodies or suppression of angiogenesis depending on the particular case [10]. Changes in the gut microbiome can be used as biomarkers for the first-time detection of CRC and as a new strategy for its prevention and treatment [11].

The development of technology has made an important contribution to the detection of bacterial and human diseases [12]. In the past, 16 S rRNA gene sequencing has been used to characterize the microbial community [13]. Currently, the use of metagenomics for whole genome sequencing is becoming increasingly popular [14, 15]. Analysis of the metagenomic dataset can reveal not only the structure of the bacterial community but also its function [16, 17].

Unfortunately, sequencing cannot distinguish between live bacteria and transient DNA [18, 19]. It is also difficult to uncover important functional elements of the gut microbiome by metagenomic alone. Therefore, it has been suggested that functional omics, such as metaproteomic and metabolomic, should also be included in the study of the gut microbiome, with “function first, taxa second” being suggested [20].

Proteomics was originally defined as the study of all proteins expressed by a single organism, which is why the analysis of the protein content of microbial communities, such as the gut microbiota, is now called “metaproteomics” [21]. Metaproteome analysis is the approach developed to identify the protein content of highly expressed genes and their pathway upon infection in an ecosystem [22, 23]. Two-dimensional polyacrylamide gel electrophoresis (2D-PAGE) followed by mass spectrometry (MS) analysis has been used for proteomics analysis in the past [24]. Nowadays, liquid chromatography/mass spectrometry (LC/MS) has become a powerful routine technology in clinical proteomics studies in protein identification, protein characterization and biomarker discovery [25].

Metabolomics is defined as the study of small molecules involved in metabolic activity from biological samples, including plasma, serum, urine, feces, and tissues [26]. The metabolome is the result of extensive interactions between gene transcription, protein expression, and environmental influences (e.g., gut microbiota). Therefore, the detection of the metabolome provides a direct readout of host physiology [27].

Nuclear magnetic resonance and MS spectrometry are the two primary metabolomics techniques (NMR). Due to its high sensitivity, high throughput, and application to a greater variety of metabolites, MS is being employed more frequently in host-microbiota research. Since biological samples are so complex, it is common practice to combine MS analysis with gas or liquid chromatography separation techniques that improve sample resolution and enable accurate identification and quantification of metabolites [28].

Nonpolar metabolites (bile acids, lipids) and polar metabolites (vitamins and their derivatives, amino acids, etc.) are the two types of metabolites that are most frequently analyzed by LC-MS [29, 30]. Untargeted metabolomics is a comprehensive analysis of all molecules that can be found in a sample, and the initial identification of thousands of substances is done through database mapping [31].

The present research was conducted to investigate taxonomic and functional changes in the gut microbiota composition, protein identification, and profile of various microbial metabolites in colorectal cancer patients. The combination of the three OMIC approaches provides a powerful new strategy for studying and validating potential screening biomarkers for cancer diagnosis and treatment.

## Materials and methods

The study was designed according to the guidelines of the research ethics committee (Reference code, 2020MH1), faculty of pharmacy, Suez Canal University, Egypt. Five females participated in the research aged from 55 to 60 years, four CRC patients were diagnosed by the oncology center, Tanta City & one healthy control after assigning informed consent. The participants were subjected to metagenomics, proteomics, and metabolomics profiles.

### Sample collection

Fresh fecal samples were collected from the participants; each participant provided one sample immediately sub-packed and marked. A sterile toothpick is used to intercept the middle part of the sample and then divided into three portions for metagenomic, metaproteomic, and metabolomics analysis. All the tubes were stored at -80˚C. The clinical and pathological characteristics of CRC patients are shown in Table 1.

**Table 1:** Clinical and pathological characteristics of CRC patients and healthy control

Sample No.	Diagnosis	Treatment
1	Colectomy + CRC	Adjuvant Folfiri protocol (folinic acid, fluorouracil (5FU) and Irinotecan Hydrochloride
2	Colectomy then Follow up (stable stage)	Adjuvant Folfox protocol (folinic acid, fluorouracil (5FU) and oxaliplatin
3	liver metastatic colorectal cancer (LMCRC)	Folfox, folfiri, combined Xelox ( Capecitabine + Oxaliplatin) and Gemcitabine
4	Colectomy and developed recurrence	Folfox, folfiri, mitomycin C, combined Xelox and Gemcitabine and Avastin (Bevacizumab) as a target therapy
5	Healthy	N/A

### Metagenomics analysis

#### DNA extraction and Bacterial whole genome sequencing

Extraction of total fecal DNA using QIAamp PowerFecal DNA Kit (cat. no. 12830-50), QIAGEN, German. All extracted DNA samples were quantified using a Qubit dsDNA kit and Nanodrop for sufficient quantity and quality of the input DNA for the shotgun. DNA sequencing was performed using the DNBSEQ platform at BGI, Hong Kong, China.

#### Bioinformatics analysis

The bioinformatics analysis started with raw sequence files. To check the quality of the reads, FASTQC was used, after that to filter the low-quality reads and adaptor sequences Trimmomatic was used for paired-end reads. Host reads were removed by kneaddata. For metagenomic phylogenetic analysis, MetaPhlAn 3.0 was used, and then produced a table containing the detected microbes and their relative abundances. The phylogenetic visualization using GraPhlAn generates circular representations of taxonomic and phylogenetic trees. For functional profiling, HUMAnN2 3.0 was used to detect the metabolic analysis network [32–35]. Its pipeline started with quality-controlled DNA sequence, and after that it is run MetaPhlAn3 to detect the microbes and get a list of microbial abundance, using bowtie2 it is detecting nucleated-level pangenome, here the algorithm is separated into two branches, the first one for mapped reads and a second one for unmapped reads. Diamond algorithm runs on unmapped reads to detect hits protein families. Both files’ core HUMAnN algorithms run on them to generate gene family abundance, pathway abundance, and pathway coverage. All of these tools used default parameters. Visualization of the highest abundant species and their pathways is done by rawgraphs [36] an open-source platform to map data dimensions onto visual variables.

#### Proteomics analysis (BGI, China)

For metaproteomics and untargeted metabolomic analysis, LC-MS/MS technology was used with high-resolution mass spectrometer Q Exactive HF (Thermo Fisher Scientific, USA).

### Sample extraction and data processing were done by BGI company, China

#### Bioinformatics analysis

The detected proteins are searched according to gene ontology (GO) annotations to study their biological function, their role in bacterial pathogenesis, and lastly to determine the effect of the proteins on the immune system using the UniProtKB database (www.uniprot.org). Potential protein pathways were identified using the KEGG database (www.genome.jp/kegg).

### Metabolomics analysis (BGI, China)

#### Data preprocessing

The mass spectrometry raw data (raw file) collected by LC-MS/MS was imported into Compound Discoverer 3.1 (Thermo Fisher Scientific, USA) for data processing, including peak extraction, retention time correction within and between groups, additive ion pooling., missing value filling, background peak labeling, and metabolite identification, and finally, information on compound molecular weight, retention time, peak area, and identification results were exported. The identification of metabolites is a combined result of BGI Library, mzCloud, and ChemSpider (HMDB, KEGG, LipidMaps) databases.

#### Metabolite extraction

Metabolite extraction was primarily performed according to previously reported methods by BGI company, Samples were analyzed on a Waters 2D UPLC coupled to a Q-Exactive heated electrospray ionization source (HESI) mass spectrometer and controlled by Xcalibur 2.3 software (Thermo Fisher Scientific, Waltham, MA, USA). Mass spectrometry adjusted for the positive/negative ionization modes.

A Log10 transfer for metaproteomics and a Log2 transfer for metabolomics is done first then to identify the differential proteins and metabolites in the control and disease groups. The fold change (FC = diseased/Healthy) is set as ≥ 1.2 as recommended by BGI Company.

## Results

### Metagenomic results

#### Taxonomic analysis

Metagenomic sequencing data of microbiota of both CRC patients and healthy person was analyzed and it was found that the gut microbiota at the taxonomic level was significantly different in CRC patients relative to healthy control. With an increase in microbiota class, order, family, genus, and species in CRC patients. There were 18 phyla in both diseased and healthy person. Notably Firmicutes phylum was the most abundant in liver metastatic colorectal cancer (LMCRC) patient and the patient developed recurrence in colon cancer after colectomy. While in other CRC patients and healthy control, Bacteroidetes was the highest abundant phylum. The Firmicutes to Bacteroidetes ratio (F/B) was as follows 0.4, 0.49, 1.46, and 1.25 in follow-up, colectomy, recurrence, and liver metastatic CRC patients, respectively. The detected genus and species in healthy control were 1122 and 3605, respectively. While 1214 genera and 4007 species were detected in CRC patients. Moreover, there were 1165 common species in both healthy and CRC patients. At the genus level, *Bacteroides*, *Eubacterium*, *Roseburia*, *Alistipes* were the most abundant genus in colectomy patient. While *Bacteroides*, *Alistipes*, and *Eubacterium* were the most abundant genus in patient in the follow-up period. In patient who developed recurrence of CRC *Bacteroides*, *Butyrivibrio*, *Dialister*, and *Eubacterium* were the most abundant genus. Moreover, *Bacteroides*, *Prevotella* then *Alistipes* were the highest abundant genus in liver metastatic CRC patient.

**Fig. 1:**
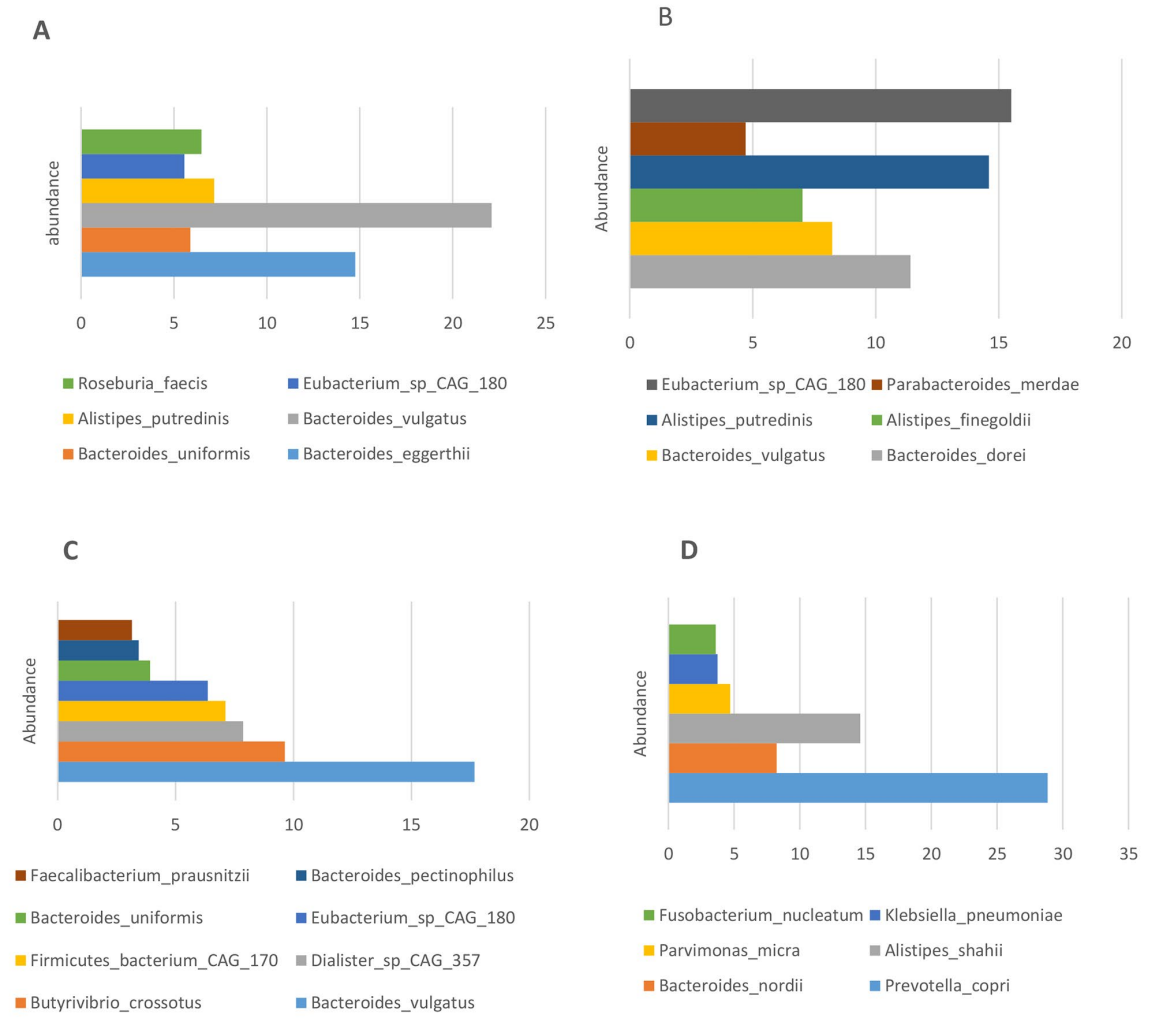
The highest abundant species in CRC patients. **A**: The Highest Abundant Species in Colectomy patient take adjuvant folfiri regimen. **B**: The Highest Abundant Species in Follow up patient. **C**: The Highest Abundant Species in patient developed recurrence of colorectal cancer. **D**: Highest abundant species in metastatic colorectal cancer patient

Concerning the species level, *Bacteroides vulgatus*, *Bacteroides eggerthii*, *Alistipes putredinis*, *Roseburia faecis*, *Bacteroides uniformis* and *Eubacterium CAG 180* were the highest abundant species in colectomy patient (Fig. 1A). *Eubacterium CAG 180*, *Alistipes putredinis*, *Bacteroides dorei*, *Bacteroides vulgatus*, *Alistipes finegoldi*, and *Parabacteroides merdaei* were the highest abundant species in the patient in the follow up period (Fig. 1B). While in patient developed recurrence of CRC, *Bacteroides_vulgatus*, *Butyrivibrio crossotus*, *Dialister CAG 357*, *Firmicutes bacterium CAG 170*, *Eubacterium CAG 180*, *Bacteroides uniformis*, *Bacteroides pectinophilus*, and *Faecalibacterium prausnitzii* were the highest abundant species (Fig. 1C). In respect to liver metastatic CRC patient, *Bacteroides nordii*, *Prevotella copri*, *Alistipes shahii*, *Klebsiella pneumoniae*, *Parvimonas micra*, and *Fusobacterium nucleatum* (Fig. 1D).

#### Functional analysis

**Fig. 2:**
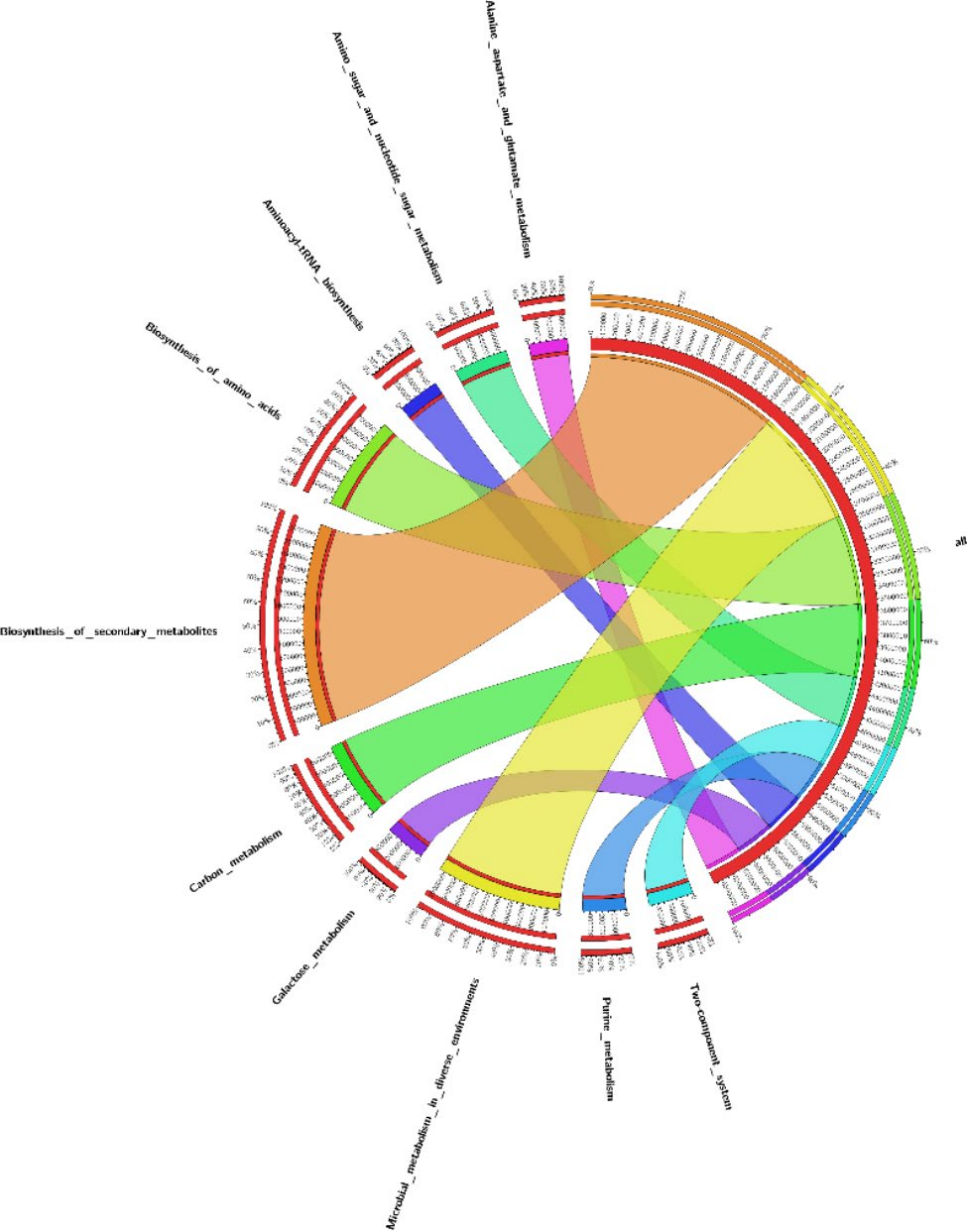
A circus plot used to express the functional annotations of the CRC patients where Outer circle: scales on samples information side is shown in percentage, indicating the ratios of functions in samples while scales on function side shows the percentage of samples that account for the same function

The functional annotations of all CRC patients detected an increase in abundance of secondary metabolites biosynthesis and microbial metabolism in diverse environments (Fig. 2). Pentose phosphate pathway was detected with high abundance in patient in recurrent state and liver metastatic CRC patient only. In addition, glycolysis and gluconeogenesis are detected only in liver metastatic CRC patient. Amino acid metabolism, TCA cycle, nucleotide biosynthesis, and glycolysis were highly detected in CRC patients.

### Proteomic results

A total of 515 proteins and 3141 peptides were found. Proteins with posttranslational modifications are excluded as they stand for human proteins. Proteins of microbial sources are of interest in this study.

Proteomic analysis was carried out using the UniProtKB database to survey the microbial community associated with colorectal cancer. The target database is microbial proteomes. Gene ontology of proteins revealed that most of these proteins are involved in different bacterial functions e.g., replication. Growth and virulence. Some of these proteins play an important role in bacterial pathogenesis and adhesion such as some uncharacterized proteins, INB domain-containing protein, Ig-like domain-containing protein, F5 8 type C domain protein, and Cadherin-like domain-containing protein. Other proteins were found to affect the immune system such as Ig-like domain- containing proteins.

Proteins from five phyla Actinobacteria, Bacteroidetes, Firmicutes, proteobacteria, and verrucomicrobia constitute 78% (281 proteins out of 360) of the microbial proteins within the human GI tract.

For the sample of the patient with follow up and taking adjuvant folfiri, it contains 146 Proteins belonging to the five phyla, which represents 40.6% of the total differential proteins from microbial source. Out of the 146 proteins, Actinobacteria constitutes 23 proteins (16%), Bacteroidetes constitute 15 proteins (10%), Firmicutes constitute 29 proteins (20%), proteobacteria constitutes 75 proteins (51%), while verrucomicrobia constitutes four proteins (3%).

For the sample of the patient with liver metastatic colon cancer patient, it contains 120 Proteins belonging to the five phyla, which represents 33.3% of the total differential proteins from microbial source. Out of the 120 proteins, Actinobacteria constitutes 19 proteins (16%), Bacteroidetes constitute 12 proteins (10%), Firmicutes constitute 24 proteins (20%), proteobacteria constitutes 62 proteins (52%), while verrucomicrobia constitutes three proteins (2.5%).

For the sample of the patient with colectomy and taking adjuvant folfox, it contains 153 Proteins belonging to the five phyla, which represents 42.5% of the total differential proteins from microbial source. Out of the 153 proteins, Actinobacteria constitutes **35** proteins (23%), Bacteroidetes constitute 12 proteins (8%), Firmicutes constitute 30 proteins (20%), proteobacteria constitutes 71 proteins (46%), while verrucomicrobia constitutes 5 proteins (3%).

For the sample of the patient with recurrence, it contains 113 Proteins belonging to the 5 phyla, which represents 31.4% of the total proteins from microbial source. Out of the 113 proteins, Actinobacteria constitutes 14 proteins (12%), Bacteroidetes constitute 14 proteins (12%), Firmicutes constitute 19 proteins (17%), proteobacteria constitutes 64 proteins (57%), while verrucomicrobia constitutes 2 proteins (2%).

Biomarkers (proteins that are only found or upregulated in a specific patient sample and not found or downregulated in other samples including the healthy one) are identified for each phylum. Biomarkers for CRC are proteins that are found in the stool of the four diseased patients but not found in the healthy one.

A total of 17 biomarker proteins belonging to phylum Actinobacteria (See Supplementary Table S1, Additional file 1), 11 biomarker proteins belonging to phylum Bacteriodetes (See Supplementary Table S2, Additional file 1), 21 biomarker proteins belonging to phylum Firmicutes (See Supplementary Table S3, Additional file 1), 48 biomarker proteins belonging to phylum proteobacteria (See Supplementary Table S4, Additional file 1) and 3 biomarker proteins belonging to phylum Verrucomicrobia were detected (See Supplementary Table S5, Additional file 1).

### Metabolomics results

The total number of metabolites according to positive (pos) and negative (neg) mode were 11,277 & 4955, respectively. While the number of metabolites with identification information was 6162 in pos mode and 2717 in neg mode. One hundred and fifty-eight metabolites were shared across four CRC females’ samples, the list of some metabolites and their expression in CRC patients were shown in Supplementary Table S6, Additional file 1. Pathways enriched by differential metabolites extracted from feces samples of CRC patients were listed in Supplementary Table S7, Additional file 1. All four patients express these compounds more than healthy one:

(Dl-glutamic acid, Pyruvic acid, Taurine, Taurocholic acid, L-serine, L-aspartic acid, L-(-)-threonine, l-methonine, (s)-malate, Dl-lysine, L-tryptophan, Dl-tryptophan, D-ornithine, Suberic acid, 7-ketodeoxycholic acid, Leucyltryptophan, Indole, Lysopc(22:1(13z)), υ-l-glutamyl-l-glutamic acid, Propionylcarnitine, (2e)-hexadecenoylcarnitine, Glycochenodeoxycholic acid, Benzaldehyde, L-pipecolic acid, P-cresol, 2’-deoxycytidine, Glycocholic acid, Phosphoric acid, Pyridoxamine, Pyridoxal, Hypoxanthine, Taurochenodeoxycholic acid, 2-keto-glutaramic acid, Leukotriene e4, 4-formyl-2-methoxyphenyl hydrogen sulfate, Indole-3-acetamide, Indole-3-acetic acid, Myristoleic acid, Eicosapentanoic acid, Indole-3-pyruvate, Vanillyl alcohol).

## Discussion

One of the most common gastrointestinal cancers is colon cancer with a high rate of distant metastasis, postoperative recurrence, and mortality. In addition, it has the second and third highest mortality rates among male and female patients, respectively [37]. The gut microbiota plays many important roles in digestion in host diseases in both immunology and tumorigenesis [38].

In the current study, the age of the participants was over 55, which means that these women are menopausal, and this is explained by the effect of female sex hormones, especially estrogen, which protects against colon cancer and regulates the composition of intestinal microbiota. Therefore, menopause is considered the main risk factor for the development of CRC in women [39].

Gut microbiota characteristics vary geographically, but several common bacterial species associated with the development of CRC are found in different populations around the world. Among them, seven enriched strains of bacteria associated with CRC were identified: *Bacteroides fragilis*; 4 oral bacterial strains of *Fusobacterium nucleatum*, *Parvimonas micra*, *Porphyromonas asaccharolytica* and *Prevotella intermedia*; *Alistipes fnegoldii* and *Termanaerovibrio acidaminovorans* [40]. In our study, *Parvimonas micra* and *Fusobacterium nucleatum* were well noted in liver metastatic CRC patients and *Alistipes fnegoldii* was found in follow-up patient.

The compositional abundance of *Alistipes* in the feces could be affected by gut dysbiosis. However, Alistipes dysbiosis can be both beneficial and detrimental, and has been linked to colon cancer [41], cardiovascular disease [42], and liver fibrosis [43]. In this study, all patients except the recurrent CRC patient contain at least one of *Alistipes* species. *Alistipes finegoldii* was a highly abundant species in patient in the follow-up period taking the folfox regimen. This finding is in agreement with Moschen et al., 2016 who showed that *Alistipes finegoldii* advocates right-sided colorectal cancer through the IL-6/STAT 3 pathway [44]. However, *Alistipes* species was also noted with high abundance in healthy person making them unuseful bacterial biomarker in our study.

*Parvimonas micra* may contribute to cancer pathogenesis, but the exact mechanism has not been fully implicit. It has also been reported that *Parvimonas micra* may be involved in gut bacterial translocation and the upregulation of interleukins in the tumor microenvironment. Furthermore, the tumor-promoting effect of *Parvimonas micra* has been reported to be associated with altered immune responses and increased inflammation in the gut [45]. *Fusobacterium nucleatum* activates β-catenin cancer pathway and alters the tumor microenvironment [46, 47]. Kostic et al., 2013 established that the abundance of *Fusobacterium nucleatum* abundance alone was insufficient as a CRC biomarker [47]. In our results, both *Parvimonas micra* and *Fusobacterium nucleatum* were highly abundant in liver metastatic CRC patients compared to healthy control, thus their synergistic presence provides a potential bacterial biomarker for CRC.

In this research, *Dialister CAG 357* species were found in patients with recurrent states. Yost et al., 2018 reported that *Dialister* was more active in the tumor sites [48]. Acetate, lactate, and propionate have been reported as metabolic end products of *Dialister*. Acetate has been reported as an important energy source for the development of solid tumors like acetate, lactate has been reported to be a key component of primary and metastatic cancer metabolism, while propionate has been reported to be an effective anti-cancer prebiotic [49–52].

*Bacteriodes* strains can affect the restoration of intestinal health in patients with inflammatory bowel disease. However, some studies show conflicting results. The effects of Bacteroides on intestinal inflammatory diseases are species or even strain-specific [53]. In our study, *Bacteriodes vulgatus* presented with high abundance in all patients except metastatic CRC patient and was not detected in healthy control. In addition, *Bacteroides eggerthii* was detected only in patient who received a chemotherapy regimen (folfiri) after colectomy. *Faecalibacterium prausnitzii and Butyrivibrio crossotus* are known butyrate producers (i.e., beneficial bacteria). In our study, they were found in patients in the follow-up period who received folfiri after colectomy and healthy control.

Firmicutes (F) and Bacteroidetes (B) phylum are considered to be major bacterial populations in the human gastrointestinal microbiome. The F/B ratio is used as an index to compare different microbial communities. A low F/B ratio has been reported to be associated with health status, whereas a high F/B ratio has been associated with CRC and obesity [54–58]. In this study, patients with recurrent and metastatic liver colon cancer had a higher proportion of Firmicutes, while the Bacteroidetes predominated in the rest of the patients and healthy control.

Concerning the functional analysis of metagenomic data in the present study, super pathway of adenosine nucleotides de novo biosynthesis I, super pathway of guanosine nucleotides de novo biosynthesis I, guanosine ribonucleotides de novo biosynthesis, adenosine deoxyribonucleotides de novo biosynthesis II and adenosine ribonucleotides de novo biosynthesis were highly abundant in all patients especially the patient in the follow up period in comparison to a healthy person. This can be explained by the fact that cancer cells require sufficient amounts of nucleotides and other macromolecules for growth and proliferation, and to meet this need, cancer cells induce the synthesis of de novo nucleotides to obtain a sufficient pool of nucleotides for nuclear acid maintenance, synthesis of proteins, conserving energy, signaling function, glycosylation mechanisms, and cytoskeletal function [59].

While pentose phosphate pathway (non-oxidative branch I) was detected with high abundant in a patient in a recurrence state and liver metastatic patient only. In addition, glycolysis I (from glucose 6-phosphate), gluconeogenesis I, super pathway of glycolysis, pyruvate dehydrogenase, TCA, and glyoxylate bypass and glycolysis III (from glucose) were detected only with high abundance in LMCRC patient. Glycolysis and gluconeogenesis are crucial pathways to the growth and survival of cancer cells and targeting these pathways as a consequence of metabolic reprogramming is an attractive therapeutic strategy [60].

The oncogenic process also relies on amino acids as building blocks for protein synthesis and as a source of energy and metabolism [61]. Cancer mainly takes up the branched chain amino acids (BCAAs) leucine, isoleucine and valine. BCAAs can be used for protein synthesis or oxidized by tumors for energy purposes [62]. In the current study, L-valine biosynthesis, L-isoleucine biosynthesis III and L-isoleucine biosynthesis I (from threonine) were detected with a high abundance in all CRC patients.

Among the major virulence factors, protein toxins of pathogenic bacteria promote colonization and proliferation within the host, which can occur in several ways, including a direct attack on DNA with genomic instability or changes in cell signals that stimulate cell proliferation and induction. Bacterial effector proteins and cell surface proteins are important factors of carcinogenic virulence for CRC development [63].

In our study, proteins from four phyla Actinobacteria, Bacteroidetes, Firmicutes and proteobacteria constituted 75% (271 proteins out of 360) of the microbial proteins within the human gastrointestinal tract.

Regarding the patient with liver metastatic CRC, it was detected that Transketolase (TKT) produced by Firmicutes bacterium was only highly expressed in this patient. The TKT contribute to the development of liver cancer by influencing bile acid metabolism, one of the enzymes in the glycolysis and pentose phosphate pathway, involved in the reprogramming of CRC metabolism, which provides raw materials for rapid cell proliferation through direct generation of ribose 5-phosphate [64]. It is well noted that, firmicutes is highly abundant in both liver metastatic CRC patient and patient who developed recurrence of colorectal cancer.

In liver metastatic colorectal cancer patient, Arginine-kinase protein was upregulated. It is expressed by Actinobacteria phylum, streptomyces genus. However, Actinobacteria phylum is relatively low abundant in follow-up patient and LMCRC patient but still higher than patient in recurrence state, colectomy patient taking folfiri regimen and healthy control. Besides that, genus, Streptomyces is well noted with low abundance in LMCRC patient only and completely absent in the remaining patient and healthy control.

Li et al., 2022 discovered that TKT expression was remarkably upregulated in colorectal cancer; abnormal high expression of TKT is associated with poor prognosis of colorectal cancer with a vital role in colorectal cancer metastasis [65].

In the present study also, Sushi domain-containing protein, Sulfide:quinone oxidoreductase, AAA domain-containing protein, AAA family ATPase and carbonic anhydrase proteins were upregulated in patients with liver metastatic CRC while it is downregulated in other samples.

Sushi domain containing 2 (SUSD2) plays a significant role in tumorigenesis, which has been identified as a regulator of colon and breast cancer. Despite that, the expression status of SUSD2 and its function in hepatocellular carcinoma remain unclear [66]. A mitochondrial sulfide-oxidizing pathway that comprises sulfide: quinone oxidoreductase (SQR) and a few other enzymes is mainly the enzymatic system that synthesizes hydrogen sulfide H2S endogenously. In cancer cells, H2S is synthesized at high levels and provokes energy metabolism and cell proliferation. In addition, H2S is known to be more persistent and overproduced with a pro-survival effect under hypoxic conditions common in the solid tumor microenvironment [67].

ATPase family AAA domain-containing protein 2 (ATAD2) has ATP binding and ATP hydrolysis activity and it is highly expressed in various cancers, including CRC. Hong et al., 2016, established that ATAD2 knockdown in colorectal cancer cell lines by RNA interference and silencing of ATAD2 inhibits migration and invasion of colorectal cancer cells by suppressing epithelial-mesenchymal transition and decreasing the activity of metalloproteinases. Making ATAD2 a novel molecular marker of metastatic colorectal cancer and providing new insights for clinical diagnosis and treatment of colorectal cancer [68].

Carbonic anhydrase protein makes reversible hydration of carbon dioxide as tumor cells need carbonic anhydrases (CAs) and many other proteins, to maintain neutral intracellular pH. During tumor growth, extracellular pH decreases and disturbs physiological processes of the surrounding normal tissue and promotes cancer growth. The study by Viikilä et al., 2016 suggests that CA II and CA XII proteins might be valuable in the assessment of CRC patients, specifically when selecting the better cancer treatment for patients [69]. In our research, it is determined that this protein is expressed by two different bacterial phyla. CAs expressed by Paenibacillus genus that belongs to Firmicutes phylum. However, this genus is only detected with very low abundance in liver metastatic colorectal cancer patient and is absent in healthy control and other patients. CAs also expressed by family flavobacteriaceae belonging to Bacteroidota phylum, this family is detected with very low abundance in both healthy control and LMCRC patient.

Regarding the patient of recurrence state, we found that The IgG Fc binding protein (FCGBP) was upregulated and downregulated in other samples. FCGBP plays an important role in tumorigenesis, progression, and prognosis, but its role in CRC metastasis remains unclear. Yuan, et al., 2021 found that FCGBP expression was much lower in liver metastatic tumor tissues compared with primary tumor tissues in liver metastatic CRC patients associated with the overall survival and progression-free survival. They found that FCGBP could predict prognosis more adequately in metastatic tumors. First reported that FCGBP can predict prognosis in liver metastatic CRC, and they thought that it could be a potential biomarker for liver metastatic CRC patients [70].

Protein kinases have a proven role in tumorigenesis and cancer progression in humans and have found targeted use in cancer therapy. By triggering the sequential activation of highly conserved protein kinases, target proteins are ultimately phosphorylated and constitute one of the most common post-translational modifications usually involved in the regulation of important cellular functions and processes, especially signal transduction [71].

In the present study, protein kinase domain-containing protein was upregulated in a patient with recurrence of CRC and downregulated in other samples. This protein is produced by *Arsenophonus endosymbiont* which belongs to the phylum proteobacteria. This protein has molecular functions including ATP binding and protein kinase activity.

Furthermore, Nucleoside diphosphate kinase protein (NME2) was found to be upregaulated in patient with recurrence state and downregulated in other samples in the present study. This protein is expressed by *Aequorivita sp. 609* which belongs to the phylum Bacteroidetes.

In addition to ATP, it plays an important role in the synthesis of nucleoside triphosphates. In a 2018 study by Wen et al., NME2 mediates 5-FU chemoresistance in CRC, and this NME2-specific inhibition restores 5-FU sensitivity in 5-FU-resistant CRC cells, reduced cell survival and increased cell apoptosis [72].

The present result showed that arylsulfatase protein is upregulated in the patient with a recurrence state while downregulated in other samples. This protein is expressed by *Lacunisphaera limnophila* species that belongs to the phylum Verrucomicrobia. It can be also expressed by *Planctomycetaceae* bacterium that belongs to the phylum Planctomycetes. Kovacs et al., 2019 reported that low expression of the arylsulfatase B (ARSB) gene may be a non-invasive indicator of CRC risk. Triple positives for maspin/ARSA/ARSB and ARSB gene expression levels < 0.5 have also been shown to indicate aggressive behavior of CRC, independent of lymph node status [73]. Notably that Verrucomicrobia phylum is detected with low abundance in patient with recurrence state of CRC.

Regarding CRC patient who passed through colectomy and received adjuvant folfox therapy, alkaline phosphatase protein (ALP) was only detected in its sample, which indicate that it may be a biomarker for CRC. This protein is expressed by Bacteroidetes bacterium. Pavkovic et al., 2015 revealed that increased level of serum ALP is associated with CRC and advise its use as early diagnostic tool for CRC [74]. In patient in the follow up period, phosphoglycerate kinase (PGK) protein was detected. PGK1 generates ATP when their supply of oxygen is limited [75]. Several malignancies including prostate cancer, breast cancer, pancreatic ductal adenocarcinoma, multidrug resistant ovarian cancer, and metastatic gastric cancer, have all been shown to exhibit an increased expression of PGK1 [76].

We also found that Acyl-CoA synthetase (ACS) is only expressed in the sample of the CRC patient who passed through colectomy, which indicates that it could be used as a biomarker for CRC. This protein is expressed by *Pseudomonas frederiksbergensis* species which belongs to phylum Proteobactria. ACS enzyme plays role in survival of cancer cells. This enzyme is an apoptosis suppressor, and its inhibition can be a good strategy to amplify etoposide antitumor effect [77].

Finally, our study showed that non-specific serine/threonine protein kinase could be used as a biomarker for CRC because it is upregulated in the four patient samples and not found in the healthy one. This protein is produced by *Sphingobacteriales bacterium*, which belongs to phylum Bacteroidetes. It has molecular functions including ATP binding, protein serine/threonine/tyrosine kinase activity, protein serine/threonine kinase activity, and protein serine kinase activity. In addition, EF-hand domain-containing protein may be a potential biomarker for CRC.

These samples exhibited nine different protein IDs standing for EF-hand domain-containing protein. They are sp|Q96A32|MLRS_HUMAN, sp|P02585|TNNC2_HUMAN, sp|P05109|S10A8_HUMAN, sp|P0DP25|CALM3_HUMAN, sp|P10916|MLRV_HUMAN, sp|P31949|S10AB_HUMAN, sp|P25815|S100P_HUMAN, sp|P63316|TNNC1_HUMAN, sp|P80511|S10AC_HUMAN. EF-hand domain-containing protein has a role in calcium ion binding. One of the EF-hand calcium binding proteins is Tescalcin (TESC) it is overexpressed in CRC but not in normal mucosa and malignant dysplastic lesions. TESC depletion reduced tumor growth in a CRC xenograft model. Therefore, TESCs are considered as potential diagnostic markers and tumor targets in colorectal cancer. In 2014, a study by Kang et al. reported elevated serum TESC levels in CRC patients, and TESC knockdown inhibited the Akt-dependent NF-κB pathway and decreased cell survival in vitro [78].

As the endpoint of the omics cascade, Metabolomics focuses on the study of whole metabolites contained in biological samples. It is currently widely used to explore the feasibility of oncotarget biomarker discovery for diagnosis, treatment and prevention based on individual tumors [79, 80]. The main bacterial genera involved in the biosynthesis of secondary bile acids are Bacteroides, Clostridium, Lactobacillus, Bifidobacterium, and Eubacterium, which also play important roles in regulating host lipid metabolism [81].

Bile acids are ordinarily produced within the liver and metabolized to secondary bile acids with the aid of the intestine microbiota in the intestinal tract, which may promote CRC pathogenesis thru the generation of genotoxic reactive oxygen species and reactive nitrogen species [82]. In our research, Taurine, Taurocholic acid, 7-ketodeoxycholic, Glycocholic acid, and Taurochenodeoxycholic acid are highly expressed in all patient. In addition, cholic acid is only highly expressed in CRC patients treated with folfiri after colectomy. While, Glycochenodeoxycholate, Glycocholate are highly expressed in CRC patients treated with folfiri after colectomy and metastatic CRC patient.

Taurocholic acid (TCA) and its components, taurine and cholic acid, support the growth of a small group of microorganisms through metabolic byproducts, are mechanistically involved in DNA damage and tumorigenesis [83]. Based on the current findings, targeting the bile acid-microbiota axis may serve as a potential therapeutic for CRC.

Polyamines are polycationic molecules required for cell growth and are biosynthesized from the amino acids’ arginine and ornithine [84]. The intestinal tract contains high levels of polyamines, including putrescine, spermidine, and spermine, obtained from food or biosynthesized by hosts and bacteria [85]. The present results showed that spermidine was highly expressed in all patients except metastatic CRC patient. In 2018, a study of urinary polyamine concentrations by Venäläinen et al. showed that patients had lower levels of diacetylspermine and spermidine after CRC curative resection [86].

In our study, all CRC patients had higher L-tryptophan expression than healthy control. There are few clinical studies of indole in CRC, and one study found an increase in indole in CRC feces, which was associated with higher concentrations in other diets [87]. Short-chain fatty acids (SCFA) are known to be significant components required for maintaining intestinal homeostasis.it is believed that their quantification in feces could serve as a biomarker for the non-invasive diagnosis of various intestinal diseases. Due to extensive absorption, only small amounts of unabsorbed SCFA (5–10% of the total) are detected in feces [88, 89]. These results are consistent with our finding, as SCFA were not detected in all samples.

Our study presented preliminary data on CRC in Egyptian patients. One of the limitations of our study is the lack of resources to select an adequate sample size to successfully address biomarkers. In conclusion, some bacterial species, proteins and metabolites can be used as CRC biomarkers. A sensitive, non-invasive and cost-effective method for the detection and recurrence of CRC may increase the therapeutic potential.

### Electronic supplementary material

Below is the link to the electronic supplementary material.

Supplementary Material 1

## Data Availability

The metagenomic shotgun data sequences of this study have been deposited in NCBI bioproject under accession number PRJNA888226 (https://www.ncbi.nlm.nih.gov/bioproject/888226). The mass spectrometry proteomics data have been deposited to the ProreomeXchange Consortium via the PRIDE [90] partner repository with the dataset identifier PXD037271.
