# Supplementary material for: Multi-omics analysis of fecal samples in colorectal cancer Egyptians patients: a pilot study
Source: BMC Microbiol. 2023 Aug 29;23:238. doi: 10.1186/s12866-023-02991-x (PMC10464353; doi:10.1186/s12866-023-02991-x)
Supplement: Supplementary file 1 — Supplementary Material 1 [file 12866_2023_2991_MOESM1_ESM.docx]

**Supplementary Table 1: Biomarkers in the phylum “Actinobacteria” named by protein ID, Protein name and species respectively.**

| Follow up | Liver metastatic CRC | colectomy+ CRC | Recurrence of CRC | Biomarkers for CRC |
| --- | --- | --- | --- | --- |
| sp\|P00558\|PGK1_HUMAN  Phosphoglycerate kinase  *Nitriliruptoraceae bacterium ZYF776* | sp\|P02765\|FETUA_HUMAN  Glycosyl hydrolase BNR repeat-containing protein  Thermomonospora curvata | sp\|P00441\|SODC_HUMAN  Superoxide dismutase [Cu-Zn]  Nitriliruptoraceae bacterium ZYF776 | sp\|Q9UM22\|EPDR1_HUMAN  Hemolysin III family protein  Streptomyces sp. SID5998 | sp\|P20142\|PEPC_HUMAN  Peptidase A1 domain-containing protein  *Nocardioides sp. BN130099* |
| sp\|P07737\|PROF1_HUMAN  Putative DUF2337 family protein  Corynebacterium epidermidicanis | sp\|P04114\|APOB_HUMAN  Uncharacterized protein  Mycolicibacterium pulveris (Mycobacterium pulveris) | sp\|P01703\|LV140_HUMAN  Uncharacterized protein  Microbacterium sp. AISO3 | sp\|P61916\|NPC2_HUMAN  Uncharacterized protein  Nocardia brasiliensis | sp\|O00584\|RNT2_HUMAN  Uncharacterized protein  *Nitriliruptoraceae bacterium ZYF776* |
| sp\|P68133\|ACTS_HUMAN  Actin, cytoplasmic 2  *Nocardioides sp. BN130099* | sp\|P06732\|KCRM_HUMAN  Arginine kinase  *Streptomyces sp. SMS_SU21* | sp\|P04155\|TFF1_HUMAN  Uncharacterized protein  Nonomuraea rubra | sp\|P62888\|RL30_HUMAN  50S ribosomal protein L30e  Nocardioides sp. BN130099 | sp\|P0DOY3\|IGLC3_HUMAN  Uncharacterized protein  *Microbacterium sp. AISO3* |
|  | sp\|P14780\|MMP9_HUMAN  Beta-N-acetylhexosaminidase  *Arthrobacter alpinus* |  | sp\|Q9GZP4\|PITH1_HUMAN  DUF2249 domain-containing protein  Mycolicibacter kumamotonensis |  |

**Supplementary Table 2: Biomarkers in the phylum “Bacteroidetes” named by protein ID, protein name and Species respectively.**

| **Follow up** | **liver metastatic CRC** | **colectomy+ CRC** | **Recurrence of CRC** | **Biomarkers for CRC** |
| --- | --- | --- | --- | --- |
| sp\|P12111\|CO6A3_HUMAN  Collagen-like protein  *Aequorivita lutea* | sp\|P00915\|CAH1_HUMAN  Carbonic anhydrase  *Aquimarina algiphila* | sp\|P16144\|ITB4_HUMAN  INB domain-containing protein  Aequorivita lutea | sp\|Q13232\|NDK3_HUMAN  Nucleoside diphosphate kinase  *Aequorivita sp. 609* | sp\|Q96PY6\|NEK1_HUMAN  Non-specific serine/threonine protein kinase  *Sphingobacteriales bacterium* |
|  | sp\|P06744\|G6PI_HUMAN  Glucose-6-phosphate isomerase  *Sporocytophaga myxococcoides* | sp\|P51161\|FABP6_HUMAN  Uncharacterized protein  Aequorivita sp. 609 |  |  |
|  | sp\|P19652\|A1AG2_HUMAN  TonB-linked outer membrane protein, SusC/RagA family  Phocaeicola coprophilus DSM 18228 = JCM 13818 | sp\|Q6P587\|FAHD1_HUMAN  Fumarylacetoacetate hydrolase family protein  Bacteroidetes bacterium |  |  |
|  | sp\|P36222\|CH3L1_HUMAN  Uncharacterized protein  *Aequorivita sp. 609* | sp\|Q9Y6X5\|ENPP4_HUMAN  Alkaline phosphatase family protein  *Bacteroidetes bacterium* |  |  |

**Supplementary Table 3: Biomarkers in the phylum “Firmicutes” named by protein ID, protein name &species respectively.**

| **Follow up** | **liver metastatic CRC** | **colectomy+ CRC** | **Recurrence of CRC** | **Biomarkers for CRC** |
| --- | --- | --- | --- | --- |
| sp\|P05787\|K2C8_HUMAN  IF rod domain-containing protein  Clostridiaceae bacterium | sp\|Q99880\|H2B1L_HUMAN  Histone domain-containing protein  *Sporolactobacillus sp. THM7-4* | sp\|Q9NSB2\|KRT84_HUMAN  IF rod domain-containing protein  *Clostridiaceae bacterium* | sp\|P01008\|ANT3_HUMAN  Serpin family protein Firmicutes bacterium |  |
| sp\|P19013\|K2C4_HUMAN  IF rod domain-containing protein  Clostridiaceae bacterium | sp\|P04040\|CATA_HUMAN  Catalase  Novibacillus thermophilus | sp\|Q8N414\|PGBD5_HUMAN  Dihydrolipoyllysine-residue succinyltransferase component of 2-oxoglutarate dehydrogenase complex  *Paenibacillus sp. NFR01* |  |  |
| sp\|Q96MH7\|CE034_HUMAN  Lactocepin  Sutcliffiella horikoshii | sp\|P04196\|HRG_HUMAN  Uncharacterized protein  Paenibacillus tianmuensis | sp\|Q9H195\|MUC3B_HUMAN  F5 8 type C domain protein  *Limosilactobacillus secaliphilus* |  |  |
| sp\|Q9BUF5\|TBB6_HUMAN  Tubulin domain-containing protein  *Sporolactobacillus sp. THM19-2* | sp\|P26447\|S10A4_HUMAN  Uncharacterized protein  *Clostridium novyi B str. NCTC 9691* | sp\|P32320\|CDD_HUMAN  Cytidine deaminase  Desulfotomaculum sp |  |  |
| sp\|Q9HC84\|MUC5B_HUMAN  LPXTG cell wall anchor domain-containing protein  *Paenibacillus sp. JW14* | sp\|P29401\|TKT_HUMAN  Transketolase (TKT)  Firmicutes bacterium | sp\|P05067\|A4_HUMAN  APP_amyloid domain-containing protein  *Tissierella creatinine* |  |  |
|  | sp\|P69905\|HBA_HUMAN  GLOBIN domain-containing protein  Staphylococcus aureus | sp\|Q12913\|PTPRJ_HUMAN  Uncharacterized protein  *Bacillus thuringiensis* |  |  |
|  | sp\|Q86WN2\|IFNE_HUMAN  Uncharacterized protein  *Neobacillus vireti LMG 21834* |  |  |  |
|  | sp\|Q9Y6N5\|SQOR_HUMAN  Sulfide:quinone oxidoreductase  *Alicyclobacillus montanus* |  |  |  |
|  | sp\|P22748\|CAH4_HUMAN  Carbonic anhydrase  Paenibacillus prosopidis |  |  |  |

**Supplementary Table 4: Biomarkers in the phylum “Proteobacteria” named by protein ID, protein name & Species respectively**

| **Follow up** | **liver metastatic CRC** | **colectomy+ CRC** | **Recurrence of CRC** | **Biomarkers for CRC** |
| --- | --- | --- | --- | --- |
| sp\|Q9Y3C5\|RNF11_HUMAN  Protein TonB  Candidimonas nitroreducens | sp\|O14531\|DPYL4_HUMAN  Dihydropyrimidinase  *Agarivorans sp. B2Z047* | sp\|Q8N2U0\|TM256_HUMAN  DUF423 domain-containing protein  Telmatospirillum siberiense | sp\|Q9Y6R7\|FCGBP_HUMAN  IgG Fc-binding protein  *Myxococcales bacterium* | sp\|P04118\|COL_HUMAN  Uncharacterized protein  *Deltaproteobacteria bacterium RIFOXYB12_FULL_58_9* |
| sp\|P02458\|CO2A1_HUMAN  Collagen-like protein  Kangiella spongicola | sp\|P01042\|KNG1_HUMAN  Nickel pincer cofactor biosynthesis protein LarC  *Polyangiaceae bacterium* | sp\|Q7L3T8\|SYPM_HUMAN  Proline-tRNA ligase  *Magnetococcales bacterium* | sp\|Q9H2K8\|TAOK3_HUMAN  Protein kinase domain-containing protein  *Arsenophonus endosymbiont of Bemisia tabaci Asia II 3* | sp\|P06312\|KV401_HUMAN  Uncharacterized protein  *Pseudoalteromonas caenipelagi* |
| sp\|P08183\|MDR1_HUMAN  ATP-binding cassette domain-containing protein  *Deltaproteobacteria bacterium* | sp\|P02042\|HBD_HUMAN  GLOBIN domain-containing protein  *Acinetobacter pittii (Acinetobacter genomosp. 3)* | sp\|O75112\|LDB3_HUMAN  LysM domain-containing protein  *Variovorax sp. CF079* |  | sp\|P0DJD9\|PEPA5_HUMAN  Saposin B-type domain-containing protein  *Salipiger sp. PrR004* |
| sp\|P21810\|PGS1_HUMAN  Bone proteoglycan II  *Kangiella spongicola* | sp\|P02675\|FIBB_HUMAN  Uncharacterized protein  Oceanospirillum sp. D5 | sp\|O75369\|FLNB_HUMAN  Uncharacterized protein  *Klebsiella pneumoniae* |  | sp\|P11142\|HSP7C_HUMAN  Molecular chaperone DnaK  *Kangiella spongicola* |
| sp\|Q53GD3\|CTL4_HUMAN  Uncharacterized protein  Candidatus Methanofishera endochildressiae | sp\|P02679\|FIBG_HUMAN  Fibrinogen C-terminal domain-containing protein  *Deltaproteobacteria bacterium HGW-Deltaproteobacteria-14* | sp\|P01599\|KV117_HUMAN  Uncharacterized protein  *Pseudoalteromonas caenipelagi* |  | sp\|P12821\|ACE_HUMAN  Peptidyl-dipeptidase  *Sorangium cellulosum (Polyangium cellulosum* |
|  | sp\|P02741\|CRP_HUMAN  Uncharacterized protein  *Pseudoalteromonas sp. NBT06-2* | sp\|P01601\|KVD16_HUMAN  Uncharacterized protein  *Pseudoalteromonas caenipelagi* |  | sp\|P63261\|ACTG_HUMAN  Actin, cytoplasmic 2  Spiribacter aquaticus |
|  | sp\|P02787\|TRFE_HUMAN  Transferrin-like domain-containing protein  *Stenotrophomonas maltophilia (Pseudomonas maltophilia) (Xanthomonas maltophilia)* | sp\|Q9UM44\|HHLA2_HUMAN  Acyl-CoA synthetase  *Pseudomonas frederiksbergensis* |  | sp\|Q9HD89\|RETN_HUMAN  Uncharacterized protein  *Proteobacteria bacterium* |
|  | sp\|P02790\|HEMO_HUMAN  Uncharacterized protein  Comamonadaceae bacterium | sp\|P04430\|KV116_HUMAN  Uncharacterized protein  *Pseudoalteromonas caenipelagi* |  | sp\|P31949\|S10AB_HUMAN  EF-hand domain-containing protein  *Stenotrophomonas maltophilia (Pseudomonas maltophilia) (Xanthomonas maltophilia)*  or  *Halobacteriovorax sp. DA5* |
|  | sp\|P04003\|C4BPA_HUMAN  Sushi domain-containing protein  Deltaproteobacteria bacterium | sp\|P12544\|GRAA_HUMAN  Trypsin  *Pseudoalteromonas sp. P1-9* |  |  |
|  | sp\|P04004\|VTNC_HUMAN  Uncharacterized protein  Deltaproteobacteria bacterium | sp\|P13639\|EF2_HUMAN  Tr-type G domain-containing protein  *Kangiella spongicola* |  |  |
|  | sp\|P05543\|THBG_HUMAN  SERPIN domain-containing protein  Desulfobacterales bacterium GWB2_56_26 | sp\|P23526\|SAHH_HUMAN  Adenosylhomocysteinase  *Gammaproteobacteria bacterium 2W06* |  |  |
|  | sp\|P0C0L5\|CO4B_HUMAN  Uncharacterized protein  *Acinetobacter baumannii* | sp\|P35030\|TRY3_HUMAN  Peptidase S1 domain-containing protein  *Nitrincola tibetensis* |  |  |
|  | sp\|P12429\|ANXA3_HUMAN  Uncharacterized protein  *Salinarimonas sp. BN140002* | sp\|P36957\|ODO2_HUMAN  Dihydrolipoyllysine-residue succinyltransferase component of 2-oxoglutarate dehydrogenase complex  *Alphaproteobacteria bacterium* |  |  |
|  | sp\|P12883\|MYH7_HUMAN  Myosin_tail_1 domain-containing protein  *Halobacteriovorax sp. DA5* | sp\|P40313\|CTRL_HUMAN  Peptidase S1 domain-containing protein  *Pseudomonas putida (Arthrobacter siderocapsulatus* |  |  |
|  | sp\|P13671\|CO6_HUMAN  Multiple EGF-like-domain protein 3  Minicystis rosea | sp\|Q01524\|DEF6_HUMAN  Uncharacterized protein  *Halomonas huangheensis* |  |  |
|  |  | sp\|Q14508\|WFDC2_HUMAN  Uncharacterized protein  *Deltaproteobacteria bacterium* |  |  |
|  |  | sp\|Q9NP55\|BPIA1_HUMAN  Uncharacterized protein  *Aquariibacter albus* |  |  |
|  |  | sp\|P01611\|KVD12_HUMAN  Uncharacterized protein  *Pseudoalteromonas caenipelagi* |  |  |

**Supplementary Table 5: Biomarkers in the phylum “Verrucomicrobia” identified by protein ID, protein name& species respectively.**

| **Follow up** | **liver metastatic CRC** | **colectomy+ CRC** | **Recurrence of CRC** |
| --- | --- | --- | --- |
|  | sp\|P02748\|CO9_HUMAN  Uncharacterized protein  Verrucomicrobiales bacterium | sp\|P10153\|RNAS2_HUMAN  Low molecular weight phosphatase family protein  *Pedosphaera sp. Tous-C6FEB* | sp\|P15289\|ARSA_HUMAN  Arylsulfatase  *Lacunisphaera limnophila* |

**Supplementary Table 6: list of metabolites and the expression of each metabolites in the four patients :**

| Metabolite Name | KEGG.ID | Super. Class | class | Subclass | CRC patient treated with folfiri after colectomy | Follow up CRC patient treated with folfox | | Liver Metastatic CRC patient | CRC recurrent patient |
| --- | --- | --- | --- | --- | --- | --- | --- | --- | --- |
| Glycine | C00037 | Organic acids and derivatives | Carboxylic acids and derivatives | Amino acids, peptides, and analogues | -2.12 | 3.10 | 2.21 | | 3.10 |
| l-phenylalanine | C00079 | Organic acids and derivatives | Carboxylic acids and derivatives | Amino acids, peptides, and analogues | 0.65 | 1.2 | 2.5 | | 1.2 |
| L-proline | C16435 | NA | null | null | 4.36 | -4.87 | 5.69 | | -4.86 |
| Leucine | C00123 | Organic acids and derivatives | Carboxylic acids and derivatives | Amino acids, peptides, and analogues | -2.85 | -1.15 | 1.57 | | -1.15 |
| Dl-glutamic acid | C00302 | Organic acids and derivatives | Carboxylic acids and derivatives | Amino acids, peptides, and analogues | 1.44 | 3.16 | 1.73 | | 3.15 |
| Taurine | C00245 | Organic acids and derivatives | Organic sulfonic acids and derivatives | Organosulfonic acids and derivatives | 3.88 | 3.04 | 2.31 | | 3.04 |
| L-asparagine | C00152 | Organic acids and derivatives | Carboxylic acids and derivatives | Amino acids, peptides, and analogues | -0.94 | 6.86 | -0.53 | | 6.86 |
| l-aspartic acid | C00049 | Organic acids and derivatives | Carboxylic acids and derivatives | Amino acids, peptides, and analogues | 1.43 | 3.15 | 1.7 | | 3.15 |
| L-histidine | C00135 | Organic acids and derivatives | Carboxylic acids and derivatives | Amino acids, peptides, and analogues | 9.89 | 6.55 | 0.29 | | 6.50 |
| L-methionine | C00073 | Organic acids and derivatives | Carboxylic acids and derivatives | Amino acids, peptides, and analogues | 4.44 | 2.48 | 1.92 | | 2.480 |
| Creatine | NA | NA | NA | NA | 4.19 | 4.55 | 0.035 | | 4.55 |
| l-tyrosine | NA | NA | NA | NA | -0.13 | 2.13 | 3.25 | | 2.13 |
| L-(-)-threonine | C00188 | Organic acids and derivatives | Carboxylic acids and derivatives | Amino acids, peptides, and analogues | 4.59 | 3.63 | 2.47 | | 3.63 |
| isoleucine | NA | NA | NA | NA | 0.08 | 4.67 | 0.17 | | 4.67 |
| dl-homoserine | NA | NA | NA | NA | -1.90 | 1.15 | -2.76 | | 1.15 |
| Dl-lysine | C16440 | Organic acids and derivatives | Carboxylic acids and derivatives | Amino acids, peptides, and analogues | 2.95 | 3.41 | 4.86 | | 3.41 |
| Dl-glutamine | NA | NA | NA | NA | 1.61 | -1.33 | 1.18 | | -1.33 |
| Choline | C00114 | Organic nitrogen compounds | Organonitrogen compounds | Quaternary ammonium salts | 1.06 | -3.49 | 3.58 | | 3.49 |
| L-tryptophan | C00078 | Organoheterocyclic compounds | Indoles and derivatives | Indolyl carboxylic acids and derivatives | 5.16 | 3.70 | 3.14 | | 3.70 |
| DL-tryptophan | C00806 | Organoheterocyclic compounds | Indoles and derivatives | Indolyl carboxylic acids and derivatives | 2.21 | 1.85 | 1.25 | | 1.85 |
| Uridine | C00299 | Nucleosides, nucleotides, and analogues | Pyrimidine nucleosides | NULL | -3.35 | -0.29 | 1.48 | | -0.29 |
| Uracil | C00106 | Organoheterocyclic compounds | Diazines | Pyrimidines and pyrimidine derivatives | 3.47 | 9.79 | 0.18 | | 9.79 |
| D-Ornithine | C00515 | Organic acids and derivatives | Carboxylic acids and derivatives | Amino acids, peptides, and analogues | 4.70 | 4.90 | 2.40 | | 4.90 |
| Ornithine | C00077 | Organic acids and derivatives | Carboxylic acids and derivatives | Amino acids, peptides, and analogues | 7.03 | 3.83 | -0.05 | | 3.83 |
| Oleic acid | C00712 | Lipids and lipid-like molecules | Fatty Acyls | Fatty acids and conjugates | -0.25 | 0.80 | 1.79 | | 0.80 |
| N-acetylserotonin | C00978 | Organoheterocyclic compounds | Indoles and derivatives | Hydroxyindoles | -6.27 | -7.46 | 2.28 | | -7.46 |
| .alpha.-aminoadipic acid | NA | Organic acids and derivatives | Carboxylic acids and derivatives | Amino acids, peptides, and analogues | -0.21 | 1.12 | -1.40 | | 1.12 |
| Suberic acid |  | Lipids and lipid-like molecules | Fatty Acyls | Fatty acids and conjugates | 1.21 | 4.71 | 1.34 | | 4.71 |
| Taurocholic acid | C05122 | NULL | NULL | NULL | 8.09 | 1.35 | 4.86 | | 1.35 |
| 7-ketodeoxycholic acid | NA | Lipids and lipid-like molecules | Steroids and steroid derivatives | Bile acids, alcohols and derivatives | 5.74 | 4.35 | 6.84 | | 4.35 |
| Sphinganine | C00836 | Organic nitrogen compounds | Organonitrogen compounds | Amines | -1.37 | -0.57 | 1.10 | | -0.57 |
| Thymine | C00178 | Organoheterocyclic compounds | Diazines | Pyrimidines and pyrimidine derivatives | -3.28 | 1.19 | -0.53 | | 1.19 |
| Leucyltryptophan | NA | Organic acids and derivatives | Carboxylic acids and derivatives | Amino acids, peptides, and analogues | 1.33 | 4.57 | 1.23 | | 4.57 |
| Kynurenic acid | C01717 | Organoheterocyclic compounds | Quinolines and derivatives | Quinoline carboxylic acids | -2.15 | 4.82 | 0.04 | | 4.82 |
| D-δ-tocopherol | C14151 | Lipids and lipid-like molecules | Prenol lipids | Quinone and hydroquinone lipids | 1.00 | 0.24 | -1.23 | | 0.24 |
| Thyronine | NA | Organic acids and derivatives | Carboxylic acids and derivatives | Amino acids, peptides, and analogues | 4.89 | -2.55 | -2.71 | | -2.55 |
| Decanoylcarnitine | NA | Lipids and lipid-like molecules | Fatty Acyls | Fatty acid esters | 0.44 | 3.52 | -2.33 | | 3.52 |
| Homocarnosine | C00884 | Organic acids and derivatives | Peptidomimetics | Hybrid peptides | -1.22 | 2.84 | 4.24 | | 2.84 |
| Indole | C00463 | Organoheterocyclic compounds | Indoles and derivatives | Indoles | 2.11 | 1.82 | 1.23 | | 1.82 |
| Lysopc(18:3(9z,12z,15z)) | NA | Lipids and lipid-like molecules | Glycerophospholipids | Glycerophosphocholines | 2.61 | -0.62 | -2.37 | | -0.62 |
| Lysopc(22:1(13z)) | NA | NULL | NULL | NULL | 2.05 | 3.44 | 1.48 | | 3.44 |
| υ-l-glutamyl-l-glutamic acid | NA | NULL | NULL | NULL | 1.44 | 5.35 | 2.19 | | 5.35 |
| D-(−)-salicin | C01451 | Organic oxygen compounds | Organooxygen compounds | Carbohydrates and carbohydrate conjugates | -9.59 | -2.17 | 1.24 | | -2.3 |
| Alanyltryptophan | NA | Organic acids and derivatives | Carboxylic acids and derivatives | Amino acids, peptides, and analogues | -0.03 | 3.80 | 0.12 | | 3.80 |
| Propionylcarnitine | NA | Lipids and lipid-like molecules | Fatty Acyls | Fatty acid esters | 1.82 | 1.95 | 1.26 | | 1.95 |
| Butenylcarnitine | NA | Organic acids and derivatives | Hydroxy acids and derivatives | Beta hydroxy acids and derivatives | -1.34 | 3.87 | 1.21 | | 3.87 |
| (2e)-hexadecenoylcarnitine | NA | Lipids and lipid-like molecules | Fatty Acyls | Fatty acid esters | 3.48 | 2.28 | 3.73 | | 2.28 |
| Benzaldehyde | C00261 | Benzenoids | Benzene and substituted derivatives | Benzoyl derivatives | 2.26 | 1.46 | 2.16 | | 1.72 |
| Glutarylcarnitine | NA | Organic acids and derivatives | Hydroxy acids and derivatives | Beta hydroxy acids and derivatives | 2.68 | 1.56 | -1.75 | | 1.89 |
| 3-hydroxyphenylacetic acid | C05593 | Benzenoids | Phenols | 1-hydroxy-4-unsubstituted benzenoids | 0.66 | 2.17 | 2.68 | | 2.17 |
| 3,4-dihydroxyphenylacetic acid | C01161 | Benzenoids | Phenols | Benzenediols | -0.81 | 5.36 | -1.04 | | -5.36 |
| Cholic acid | C00695 | Lipids and lipid-like molecules | Steroids and steroid derivatives | Bile acids, alcohols and derivatives | 1.85 | -0.18 | -0.51 | | -0.18 |
| L-pipecolic acid | C00408 | Organic acids and derivatives | Carboxylic acids and derivatives | Amino acids, peptides, and analogues | 2.97 | 3.42 | 4.87 | | 3.42 |
| P-cresol | C01468 | Benzenoids | Phenols | Cresols | 3.60 | 2.07 | 2.25 | | 2.07 |
| Phthalic acid | C01606 | Benzenoids | Benzene and substituted derivatives | Benzoic acids and derivatives | -4.17 | -0.16 | 1.60 | | -0.16 |
| Phenylacetic acid | C07086 | Benzenoids | Benzene and substituted derivatives | NULL | 1.18 | 1.21 | -0.19 | | 1.21 |
| Spermidine | C00315 | Organic nitrogen compounds | Organonitrogen compounds | Amines | 1.91 | 7.23 | 0.17 | | 7.23 |
| Ophthalmic acid | C21016 | Organic acids and derivatives | Carboxylic acids and derivatives | Amino acids, peptides, and analogues | 8.44 | 1.32 | -0.62 | | 1.32 |
| 2'-deoxyadenosine | C00559 | Nucleosides, nucleotides, and analogues | Purine nucleosides | Purine 2'-deoxyribonucleosides | -4.45 | -3.10 | 1.93 | | -3.10 |
| 5'-methylthioadenosine | C00170 | Nucleosides, nucleotides, and analogues | 5'-deoxyribonucleosides | 5'-deoxy-5'-thionucleosides | 4.01 | 1.58 | -2.19 | | 1.58 |
| 2'-deoxycytidine | C00881 | Nucleosides, nucleotides, and analogues | Pyrimidine nucleosides | Pyrimidine 2'-deoxyribonucleosides | 4.78 | 1.28 | 2.85 | | 2.05 |
| N-acetylcytidine |  | Nucleosides, nucleotides, and analogues | Pyrimidine nucleosides | NULL | 0.12 | 3.96 | 0.11 | | 3.96 |
| 5-methyldeoxycytidine |  | Nucleosides, nucleotides, and analogues | Pyrimidine nucleosides | Pyrimidine 2'-deoxyribonucleosides | -2.08 | 1.28 | -2.24 | | 1.28 |
| N-phenylacetylglutamine | C04148 | Organic acids and derivatives | Carboxylic acids and derivatives | Amino acids, peptides, and analogues | 4.23 | 0.25 | -0.96 | | 0.25 |
| Glycochenodeoxycholate | C05466 | Null | null | null | 3.62 | -0.02 | 6.90 | | -0.02 |
| Glycocholate | C01921 | Null | null | null | 6.74 | 0.13 | 7.36 | | 0.13 |
| Glycocholic acid | Null | Lipids and lipid-like molecules | Steroids and steroid derivatives | Bile acids, alcohols and derivatives | 2.84 | 1.79 | 4.90 | | 1.79 |
| Phosphoric acid | C00009 | Homogeneous non-metal compounds | Non-metal oxoanionic compounds | Non-metal phosphates | 5.78 | 2.34 | 3.79 | | 2.34 |
| 8-hydroxy-deoxyguanosine | NA | Nucleosides, nucleotides, and analogues | Purine nucleosides | Purine 2'-deoxyribonucleosides | -0.27 | 5.33 | 4.68 | | 4.63 |
| Leukotriene e4 | NA | Organic nitrogen compounds | Organonitrogen compounds | Amines | 5.05 | 1.90 | 1.58 | | 1.90 |
| Pyridoxamine | C00534 | Organoheterocyclic compounds | Pyridines and derivatives | Pyridoxamines | 6.81 | 4.12 | 6.90 | | 4.12 |
| Hypoxanthine | C00262 | Organoheterocyclic compounds | Imidazopyrimidines | Purines and purine derivatives | 1.75 | 3.06 | 1.73 | | 3.06 |
| Hippuric acid | C01586 | Benzenoids | Benzene and substituted derivatives | Benzoic acids and derivatives | -1.95 | 0.001 | 2.05 | | 0.001 |
| Arachidonic acid | C00219 | Lipids and lipid-like molecules | Fatty Acyls | Fatty acids and conjugates | -4.52 | 1.92 | -0.77 | | 1.92 |
| Traumatic acid | C16308 | Lipids and lipid-like molecules | Fatty Acyls | Fatty acids and conjugates | -2.88 | 1.30 | 1.77 | | 1.30 |
| inosine-5'-monophosphate (imp) | NA | NA | NA | NA | 2.47 | 3.30 | 0.03 | | 3.30 |
| Uric acid | C00366 | Organoheterocyclic compounds | Imidazopyrimidines | Purines and purine derivatives | 10.91 | 0.55 | -0.69 | | 0.55 |
| Chenodeoxycholic acid 3-sulfate | NA | Lipids and lipid-like molecules | Steroids and steroid derivatives | Bile acids, alcohols and derivatives | 5.25 | 0.09 | -0.85 | | 3.25 |
| Indole-3-acetaldehyde | C00637 | Organoheterocyclic compounds | Indoles and derivatives | Indoles | -0.67 | 1.50 | 1.83 | | 1.50 |
| Indole-3-acetamide | C02693 | Organoheterocyclic compounds | Indoles and derivatives | Indoles | 1.83 | 5.65 | 2.91 | | 5.65 |
| Indole-3-acetic acid | C00954 | Organoheterocyclic compounds | Indoles and derivatives | Indolyl carboxylic acids and derivatives | 1.65 | 4.15 | 2.96 | | 4.15 |
| Indole-3-ethanol | C00955 | Organoheterocyclic compounds | Indoles and derivatives | Indoles | 1.28 | 0.01 | -1.58 | | 0.01 |
| Indole-3-pyrubate | C00331 | Organoheterocyclic compounds | Indoles and derivatives | Indolyl carboxylic acids and derivatives | 4.23 | 4.17 | 1.56 | | 4.17 |
| Adenine | C00147 | Organoheterocyclic compounds | Imidazopyrimidines | Purines and purine derivatives | 2.49 | 10.58 | -0.17 | | 10.58 |
| Xanthine | C00385 | Organoheterocyclic compounds | Imidazopyrimidines | Purines and purine derivatives | -5.73 | 1.82 | 1.76 | | 1.82 |
| Taurochenodeoxycholic acid | C05465 | Lipids and lipid-like molecules | Steroids and steroid derivatives | Bile acids, alcohols and derivatives | 4.62 | 2.01 | 1.78 | | 2.01 |
| Vanillyl alcohol | C06317 | Benzenoids | Phenols | Methoxyphenols | 1.91 | 3.83 | 1.82 | | 3.83 |
| 8-hydroxy-deoxyguanosine | NA | Nucleosides, nucleotides, and analogues | Purine nucleosides | Purine 2'-deoxyribonucleosides | -0.27 | 5.33 | 4.68 | | 5.33 |
| Pyridoxal | C00250 | Organoheterocyclic compounds | Pyridines and derivatives | Pyridine carboxaldehydes | 3.89 | 2.95 | 2.07 | | 2.95 |
| 2-keto-glutaramic acid | C00940 | Organic acids and derivatives | Keto acids and derivatives | Short-chain keto acids and derivatives | 2.35 | 2.85 | 1.22 | | 2.85 |
| Eicosapentanoic acid | C06428 | Lipids and lipid-like molecules | Fatty Acyls | Fatty acids and conjugates | 1.45 | 4.24 | 1.24 | | 4.24 |
| 4-formyl-2-methoxyphenyl hydrogen sulfate | NA | Organic acids and derivatives | Organic sulfuric acids and derivatives | Arylsulfates | 3.47 | 2.85 | 2.76 | | 2.85 |
| Myristoleic acid | NA | Lipids and lipid-like molecules | Fatty Acyls | Fatty acids and conjugates | 1.85 | 4.41 | 3.61 | | 4.41 |
| Gamma-aminobutyric acid | C00334 | Organic acids and derivatives | Carboxylic acids and derivatives | Amino acids, peptides, and analogues | -3.92 | 1.20 | 0.54 | | 0.20 |
| Citrate | C00158 | Organic acids and derivatives | Carboxylic acids and derivatives | Tricarboxylic acids and derivatives | 6.61 | -0.76 | -1.34 | | 5.21 |
| L-serine | C00065 | Organic acids and derivatives | Carboxylic acids and derivatives | Amino acids, peptides, and analogues | 7.18 | 5.06 | 4.21 | | 2.44 |
| Pyruvic acid | C00022 | Organic acids and derivatives | Keto acids and derivatives | Alpha-keto acids and derivatives | 5.4 | 2.44 | 2.46 | | 3.85 |
| (s)-malate | C00149 | Organic acids and derivatives | Hydroxy acids and derivatives | Beta hydroxy acids and derivatives | 3.38 | 2.07 | 1.28 | | 2.26 |
| 3b-hydroxy-5-cholenoic acid | NA | Lipids and lipid-like molecules | Steroids and steroid derivatives | Bile acids, alcohols and derivatives | -4.75 | 2.58 | 4.97 | | 3.25 |
| Glycochenodeoxycholic acid | NA | NA | NA | NA | 2.81 | 1.39 | 5.87 | | 3.29 |

**Supplementary Table 7: Pathway enrichment analyses based on the differential metabolites of colorectal cancer:**

| Name of pathways | CRC patient treated with folfiri after colectomy | Follow up CRC patient treated with folfox | Liver Metastatic CRC patient | CRC recurrent patient |
| --- | --- | --- | --- | --- |
| Alanine, aspartate and glutamate metabolism | + | + | + | + |
| Aminoacyl-tRNA biosynthesis | + | + | + | + |
| Arginine and proline metabolism | + | + | + | + |
| Cyanoamino acid metabolism | + | + | + | + |
| Cysteine and methionine metabolism | + | + | + | + |
| Glycine, serine and threonine metabolism | + | + | + | + |
| Nitrogen metabolism | + | + | + | + |
| Pantothenate and CoA biosynthesis | + | + | + | + |
| Taurine and hypotaurine metabolism | + | + | + | + |
| Valine, leucine and isoleucine biosynthesis | + | + | + | + |
| Propanoate metabolism | + | - | - | - |
| Sulfur metabolism | + | + | + | + |
| beta-Alanine metabolism | + | + | + | + |
| D-Glutamine and D-glutamate metabolism | + | + | + | + |
| Purine metabolism | + | + | + | + |
| Pyrimidine metabolism | + | + | + | + |
| Glycolysis or Gluconeogenesis | + | + | + | + |
| Glutathione metabolism | + | + | + | + |
| Butanoate metabolism | + | + | + | + |
| Valine, leucine and isoleucine degradation | + | + | + | + |
| *(-) not present ; (+) present | | | | |
